# The relationship between telework from home and employee health: a systematic review

**DOI:** 10.1186/s12889-021-12481-2

**Published:** 2022-01-07

**Authors:** Lars-Kristian Lunde, Lise Fløvik, Jan Olav Christensen, Håkon A. Johannessen, Live Bakke Finne, Ingrid Løken Jørgensen, Benedicte Mohr, Jolien Vleeshouwers

**Affiliations:** 1 Department of Occupational Medicine and Epidemiology, National Institute of Occupational Health, Oslo, Norway; 2 Department of Work Psychology, National Institute of Occupational Health, Oslo, Norway

**Keywords:** Working from home, E-work, Satellite work, remote work, General health, Stress, Well-being, Exhaustion, Burnout, Pain, Life satisfaction, Leisure satisfaction

## Abstract

**Background:**

Globalization and technological progress have made telework arrangements such as telework from home (TWFH) well-established in modern economies. TWFH was rapidly and widely implemented to reduce virus spread during the Coronavirus disease (COVID-19) pandemic, and will probably be widespread also post-pandemic. How such work arrangements affect employee health is largely unknown. Main objective of this review was to assess the evidence on the relationship between TWFH and employee health.

**Methods:**

We conducted electronic searches in MEDLINE, Embase, Amed, PsycINFO, PubMed, and Scopus for peer-reviewed, original research with quantitative design published from January 2010 to February 2021. Our aim was to assess the evidence for associations between TWFH and health-related outcomes in employed office workers. Risk of bias in each study was evaluated by the Newcastle-Ottawa Scale and the collected body of evidence was evaluated using the the Grading of Recommendations Assessment, Development and Evaluation (GRADE) approach.

**Results:**

We included 14 relevant studies (22,919 participants) reporting on 28 outcomes, which were sorted into six outcome categories (general health, pain, well-being, stress, exhaustion & burnout, and satisfaction with overall life & leisure). Few studies, with many having suboptimal designs and/or other methodological issues, investigating a limited number of outcomes, resulted in the body of evidence for the detected outcome categories being GRADED either as low or very low.

**Conclusions:**

The consisting evidence on the relationship between TWFH and employee health is scarce. The non-existence of studies on many relevant and important health outcomes indicates a vast knowledge gap that is crucial to fill when determining how to implement TWFH in the future working life.

**Systematic review registration number:**

PROSPERO registration ID # CRD42021233796.

**Supplementary Information:**

The online version contains supplementary material available at 10.1186/s12889-021-12481-2.

## Background

Driven by globalization, digitalization, and technological progress the international working life has gone through remarkable transitions during the previous decades. Generally, this transition has changed both the content of work and how it is organized and carried out [1]. Among other types of flexible work arrangements, telework is now well-established as a work arrangement in modern economies, where employees are not located at a central office building, but rather work at a distant location [2]. Telework is a subcategory of the broader concept remote work, with the additional distinction that telecommunication technology is used to replace the physical commute to work [3]. Telework arrangements were first made practically feasible in the early 80s due to technological progress, and have since slowly become more widespread [2, 4]. In 2015, 17% of European workers were engaged in some form of telework [5]. However, as a result of the Coronavirus disease (COVID-19) pandemic that hit the world fully in 2020, these types of work arrangements were rapidly and widely implemented to reduce virus spread. After national restrictions were introduced, 37% of all workers in the EU carried out their work from a remote location, with numbers as high as 50-60% in the Nordic countries [6]. An important question is how this affects the employee, considering the possibility that systems and work arrangements introduced as a result of the COVID-19 will to some degree remain part of future working life.

It is plausible that this new way of arranging work can disrupt work environment and health. For instance, both physical and psychosocial working conditions are patently different when comparing working from an office location to teleworking. Hence, up to date knowledge is necessary to clarify if and how a shift towards telework impact employee health. It is commonly agreed that employment, the characteristics of an employee’s work, and the workplace itself may influence the individual’s health [7–9]. Such relationships between one’s job and health may work through psychosocial, organizational, or physical mechanisms. A shift towards teleworking could for instance impact the feeling of social connectedness and support from leaders and colleagues or other psychological aspects of the job, which are known to be important for physical and mental health [9–12]. Further, it is possible that more flexible work arrangements may alter the relationship between demand and control, known to predict various health outcomes [13–17]. Similarly, work autonomy is also well-documented to have impact on health [18]. However, we do not know how telework affects flexibility and autonomy, how it is connected to employees’ psychological job demands and job control, nor how it impacts employee health. Moreover, telework may also influence the line between work and private life or alter physical and ergonomic characteristics or other issues related to health, environment, and safety at work, compared to a regular office setting [19].

With the increase seen in telework arrangements the last decades, a trend plausibly accelerated by the COVID-19 pandemic in the years to come, it is of major importance to be aware of how the move towards such work arrangements may affect employee health. To increase relevance and limit heterogeneity in type of telework in this review, we exclusively investigate teleworkers that are teleworking from home (TWFH) [3]. Our aim was to systematically review the evidence from studies investigating the association between TWFH and employees’ physical and mental health.

## Methods

### Protocol registration

The present systematic review is part of a larger study with protocol registered in the international register for systematic reviews, PROSPERO (ID # CRD42021233796). This also includes a systematic review on work environmental outcomes. Thus, we carried out a combined search and study selection with a final separation into two distinct systematic reviews, where findings of the health-related outcomes will be presented here. The review was carried out following standardized procedures and is reported following the Preferred Reporting Items for Systematic Reviews and Meta-Analyses (PRISMA) guidelines [20].

### Selection criteria

We aimed to include all relevant original research studies with a quantitative design. Studies had to be written in English, internationally published, and peer-reviewed. We did not include studies with purely qualitative design, studies only reporting descriptive statistics, systematic reviews with or without meta-analyses, dissertations, book chapters, or theoretical work such as editorials, short communications, or conference abstracts.

Further, we included populations consisting of employed workers mainly conducting office work. We excluded studies of self-employed and/or students. The exposure of interest was TWFH. Thus, we excluded studies where exposure were not distinct measures of TWFH, e.g. availability of telework programs, organizational support for telework, telework where site was not specified, or flexible work where TWFH was not specified. We did also exclude studies where restrictions due to COVID-19 were so strict (e.g. curfew) it was reason to believe that the effect of this would significantly bias an actual effect of TWFH. We did not exclude outcomes based on method of measurement (e.g. diagnosis, registers, self-reports), and thus, aimed to include all types of physical and mental health outcomes.

### Search strategy and study selection

Using a range of relevant variations of free text terms, the following databases were searched for relevant literature September 23rd, 2020: MEDLINE, Embase, Amed, PsycINFO, PubMed, and Scopus. The search was conducted by trained research librarians and was restricted to include publications from and including 2010 and up to the search date. A full description of the search terms used for each of the databases is found in the supplementary (Supplementary S1). We conducted a second similar search February 26th, 2021 to include studies published between this date and the date of the initial search. After duplicate removal, each retrieved publication was screened by two researchers, individually and blinded for each other’s decision. We based eligibility on the selection criteria, with a selection process of two separate and sequencing steps; first from title and abstract, and thereafter by reading full-text articles. Disagreements were solved through discussion between the two involved researchers, or if they could not agree, by including a third researcher carrying out an individual evaluation. We did manual searches of the reference lists of all papers included after full-text screening, with potential candidates going through an identical screening process. We used Covidence® software [21] to manage articles during the selection process.

### Data extraction

We used a pre-defined data extraction sheet with instructions to facilitate data extraction. All involved researchers met prior to data extraction to ensure consensus. Variables extracted included, but were not limited to: exposure details and instruments used to sample, outcome measures of health and instruments used to sample, study design, country of origin, population occupation, sample size, response rate, attrition, control variables (if applicable), and main results with mediators and moderators (if applicable).

### Risk of bias and quality of evidence

Risk of bias (ROB) for each single study and its relevant outcome(s), was evaluated by the Newcastle-Ottawa Scale (NOS) [22] by two individual researchers blinded for each other’s initial rating. We chose the NOS tool as it has been developed to assess the quality of non-randomized studies for the purpose of inclusion in systematic reviews [22, 23]. NOS assesses ROB within three domains: study group selection, group comparability, and the ascertainment of either the exposure or outcome of interest. Each domain is awarded stars according to its ROB, where appointed star(s) equals lower ROB and no stars equals higher ROB. Based on study design we used forms developed for cohort and cross-sectional studies and converted the number of stars to a grading of poor, fair, or good quality. See supplementary for thresholds for conversion of NOS into the different categories (Supplementary S2). We added the possibility to obtain one star also for self-reported outcomes from structured surveys, based on the non-viable option to measure several of the relevant health outcomes by independent blind assessment or record linkage (e.g. symptoms that are inherently subjective, such as pain and/or discomforts, exhaustion, well-being). Additionally, questions on exposure from structured surveys were considered in the category of structured interview and obtained one star. Rating conflicts were solved through discussion between the two involved researchers, or when necessary, by involving a third researcher.

The overall body of evidence for each category of outcomes (similar outcomes from all available studies) was then evaluated by three researchers using the Grading of Recommendations Assessment, Development and Evaluation (GRADE) [24] and the GRADEpro® software [25]. Thus, the quality of evidence of the studies reporting on similar outcomes was evaluated combined and received one of four scores; very low, low, moderate, or high. We did not consider publication bias by e.g. funnel plots due to data characteristics and the limited number of studies for each outcome.

### Data synthesis

Due the expected heterogenous nature of exposure assessments and outcome measures we did not carry out a meta-analysis. As such, the methodological differences were considered too large to justify any pooling of data. Thus, characteristics and summary of results from each individual study were first extracted and described. Thereafter, each respective health outcome of interest was grouped together with similar outcomes from other studies to form outcome categories. Finally, we evaluated the overall certainty of evidence for each individual outcome category [26].

## Results

### Study selection

**Fig. 1:**
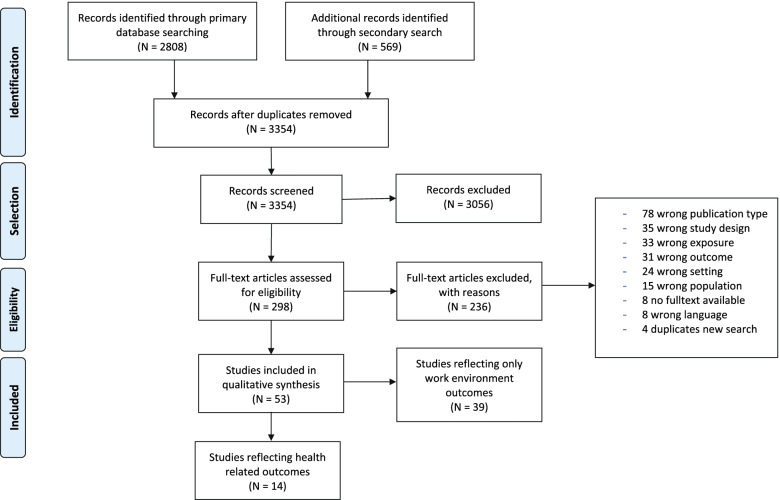
PRISMA flow diagram

The PRISMA flow diagram in Fig. 1 shows the study selection process. The initial search of the databases identified 2808 records, while the updated search identified an additional 569 records. After duplicate removal, 3354 records were screened based on title and abstract, and thereafter the remaining 298 records were screened based on full-text. Of these, 53 studies were considered satisfying according to our selection criteria. Thirty-nine studies reported only on work environment-related outcomes and will be reported elsewhere, while 14 reported on one or more health-related outcomes and were included in this review.

### Study descriptives

The 14 included studies reported on 28 outcomes. Six studies reported on several outcomes, five of these reported outcomes in more than one outcome category. We identified and constructed six outcome categories, where three studies reported on general health (three outcomes in total), two studies reported on pain (two outcomes in total), four studies reported on well-being (eight outcomes in total), six studies reported on stress (six outcomes in total), six studies reported on exhaustion and burnout (six outcomes in total), and two studies reported on satisfaction with overall life and leisure (three outcomes in total). See Table 1 for number and design of studies reporting on outcomes in each outcome category, and total outcomes included in each category. See supplementary S3 for an overview of which outcome category outcomes from each study were placed in. Eight of the 14 included studies had cross-sectional design, while six had longitudinal designs (follow-up from 3 months to 10 years). Nine studies originated from USA, four from Europe (two from Belgium, one from Germany, and one from Great Britain) and one study originated from Africa (South Africa). Sample size varied from 51 to 6132, and 53% the investigated workers were employed in the USA. Female proportion in samples ranged from 17 to 69% and age ranged from 16 to 65. All exposure and outcome measures were based on self-report. See Table 2 for study characteristics and findings.

**Table 1:** Number and design of studies reporting on outcomes in each outcome category

Outcome category	No. outcomes in category	Cross-sectional studies in category	Longitudinal studies in category	RCT studies in category
General health^a^	3	0	3	0
Pain^b,c^	2	2	0	0
Well-being^b,c^	8	3	1	0
Stress^b,c,d^	6	5	1	0
Exhaustion and burnout^b,c,d^	6	5	1	0
Satisfaction life and leisure^a^	3	0	2	0

^a^Kroll & Nuesch [27], and Reusche 2019 [28] reported on outcomes in the outcome categories general health and satisfaction with overall life and leisure

^b^Song & Gao [29] reported on outcomes in the outcome categories well-being, pain, stress, and exhaustion and burnout

^c^Giménez-Nadal et al. [30] reported on pain, well-being, stress, and exhaustion and burnout

^d^Vander Elst et al. [31] reported on outcomes in the outcome categories stress and exhaustion and burnout

**Table 2:** Study characteristics and findings

Study	Population	N	Exposure measure	Outcome measure(s)	Outcome group	Design	Follow-up	Findings	Quality
Anderson et al. 2015 [32]	Age: ≤65 yrs. Gender: 50% female Type of work: government agency Country: USA	102	TWFH or office Self-report	Negative/positive affective well-being Self-reported, 10 items, 5-point Likert-scale	Well-being	Cross-sectional	N.A.	TWFH was associated with more positive affective well-being and less negative affective well-being on days TWFH	Poor
Baard & Thomas 2010 [33]	Age: ≥23 yrs. Gender: 46% female Type of work: telecommunication and finance Country: South Africa	63	TWFH ≥1d/wk., but less than 5d/wk. Self-report	Stress Self-reported 5-point Likert-scale	Stress	Cross-sectional	N.A.	Participants reported decreased stress when TWFH, with those having ≥3 dependents at home more often reporting a decrease in stress levels	Poor
Delanoeije & Verbruggen 2020 [34]	Age: 24-65 yrs. Gender: 24% female Type of work: engineering and estimating within construction and property Country: Belgium	64	TWFH or office. Intervention group TWFH ≤2d/wk. vs control only working at the office	Stress Self-reported, 5 items, 7-point Likert-scale	Stress	Quasi-experimental (longitudinal)	3 months	There was no difference in stress between intervention groups, but workers TWFH reported lower levels of stress on days TWFH compared to days working at the office	Good
Fonner & Roloff 2012 [35]	Age: 39 yrs. (mean) Gender: 54% female Type of work: not specified office work Country: USA	193	High-intensity TWFH, ≥3d/wk. or office-based Self-reported	Stress from interruptions Self-reported, 6-item scale	Stress	Cross-sectional	N.A.	Office-based employees reported significantly higher levels of stress from interruptions compared to those TWFH, but are more negatively affected by interruptions per se	Poor
Giménez-Nadal et al. 2020 [30]	Age: 16-65 yrs. Gender: 45% female Type of work: general working population Country: USA	2471	TWFH or office Self-reported	Pain, happiness, sadness, tiredness, stress Self-reported, single-items, 7-point Likert-scale	Pain, Well-being, Exhaustion & burnout, Stress	Cross-sectional	N.A.	Male workers TWFH reported less stress, pain and tiredness compared to office-based workers. No associations were found for happiness or sadness. No associations were found between TWFH and any outcome for female workers	Fair
Henke et al. 2016 [36]	Age: 18-64 yrs. Gender: 58% female Type of work: Finance Country: USA	3703	Hours logged TWFH	Edington risk score Self-reported, Health risk assessment	General health	Longitudinal	1 year	Employees TWFH had an overall reduced risk of developing health problems	Good
Hoffman et al. 2020 [37]	Age: not provided Gender: 69% female Type of work: radiation oncology Country: USA	575	TWFH some or all of the time Self-reported	Burnout Self-reported, single item, 5-point Likert-scale	Exhaustion and burnout	Cross-sectional	N.A.	The majority of employees TWFH reported this as positive experience, a feeling associated with reduced burnout	Poor
Kroll & Nüesch 2019 [27]	Age: 20 – 60 yrs. Gender: 47% female Type of work: general working population Country: Germany	6132	Carrying out telework at home or not Self-reported	Perceived health, leisure satisfaction Self-reported, single item, 5 and 10-point Likert-scale	General health, satisfaction with life and leisure	Longitudinal	10 yrs.	TWFH was not associated with leisure satisfaction or perceived health	Good
Reusche D. 2019 [28]	Age: 18 – 64 yrs. Gender: 48% female Type of work: general working population Country: Great Britain	3738	TWFH or not Self-reported	Satisfaction with life, leisure time, and health Self-reported, single-items, 7-point Likert-scale	General health, satisfaction with life and leisure	Longitudinal	7 yrs.	TWFH was associated with increased satisfaction with leisure time, but not with overall life or health	Good
Sardeshmukh et al. 2012 [38]	Age: ≥26 yrs. Gender: 29% female Type of work: logistics Country: USA	417	TWFH part time Self-reported	Exhaustion Self-reported, 8-item scale	Exhaustion and burnout	Cross-sectional	N.A.	TWFH was associated lower degree of exhaustion, mediated by role conflict, role ambiguity, time pressure, support, feedback, and autonomy	Poor
Shepherd- Banigan et al. 2016 [39]	Age: 18 – 43 yrs. Gender:100% female Type of work: general working population Country: USA	570	TWFH or not Interview	Symptoms of depression Self-reported, 20-items, 3-point Likert-scale	Well-being	Longitudinal	2 yrs.	TWFH was related to less symptoms of depression in women with young children (≤24 months) who returned to work within 6 months after childbirth	Good
Song & Gao 2020 [29]	Age: 18 – 65 yrs. Gender: 41% female Type of work: general working population Country: USA	3962	TWFH or office Self-reported	Pain, happiness, sadness, meaningfulness, stress, tiredness Self-reported, single-items, 7-point Likert-scale	Pain, Well-being, Stress, and Exhaustion & burnout	Cross-sectional	N.A.	Overall, there was no association between TWFH and pain, tiredness, happiness, sadness or meaningfulness. There was an increase in stress for fathers TWFH and a reduction in happiness for mothers TWFH. TWFH was associated with higher stress levels.	Good
Vander Elst et al. 2017 [31]	Age: 45 yrs. (mean) Gender: 17% female Type of work: telecommunication Country: Belgium	878	Extent of TWFH Self-reported	Emotional exhaustion, Cognitive stress complaints Self-reported, single-items, 7- and 5-point Likert-scales	Stress, Exhaustion & Burnout	Cross-sectional	N.A.	No associations between TWFH and emotional exhaustion and cognitive stress complaints. Results showed indirect relationships via level of felt social support for both outcomes.	Good
Windeler et al. 2017^a^ [40]	Age: 43 yrs. (mean) Gender: 39% female Type of work: information technology Country: USA	51	TWFH 1-2 d/wk. Self-reported	Work exhaustion Self-reported, 4-items, 7-point Likert-scale	Exhaustion & Burnout	Longitudinal	4 months	TWFH 1-2 d/wk. was associated with reduced work exhaustion due to interpersonal interaction, but with increased work exhaustion related to external interaction. TWFH did not affect the relationship between interdependence and work exhaustion. Men and older workers experienced higher levels of exhaustion after beginning to TWFH.	Good

^a^Windeler et al. [40] report on two studies, where we report only study 1 (within subject assessment with longitudinal design)

### Findings for health-related outcome categories

See Table 2 for individual study results and Table 3 for overall body of evidence.

**Table 3:** Overall body of evidence

Outcome category	Findings for cross-sectional studies			Findings for longitudinal studies			Overall certainty of evidence (GRADE)^**a**^
	Poor	Fair	Good	Poor	Fair	Good	
General health							Very low
*Beneficial*	‧	‧	‧	‧	‧	1	
*No association*	‧	‧	‧	‧	‧	2	
*Detrimental*	‧	‧	‧	‧	‧	‧	
Pain							Very low
*Beneficial*	‧	1^b^	‧	‧	‧	‧	
*No association*	‧	‧	1	‧	‧	‧	
*Detrimental*	‧	‧	‧	‧	‧	‧	
Well-being							Very low
*Beneficial*	1	‧	‧	‧	‧	1^c^	
*No association*	‧	1	1	‧	‧	‧	
*Detrimental*	‧	‧	‧	‧	‧	‧	
Stress							Low
*Beneficial*	2	1^d^	‧	‧	‧	‧	
*No association*	‧	‧	1	‧	‧	1^e^	
*Detrimental*	‧	‧	1	‧	‧	‧	
Exhaustion & Burnout							Very low
*Beneficial*	2	1^f^	‧	‧	‧	‧	
*No association*	‧	‧	2	‧	‧	1^g^	
*Detrimental*	‧	‧	‧	‧	‧	‧	
Satisfaction life & leisure							Low
*Beneficial*		‧	‧	‧	‧	1^h^	
*No association*		‧	‧	‧	‧	1	
*Detrimental*		‧	‧	‧	‧	‧	

^a^None of the outcome groups were upgraded due to magnitude of effect, dose-response, or confounding

^b^for males

^c^for females with children ≤2 years

^d^for males

^e^no difference between groups, but those TWFH reported lower levels of stress on days TWFH

^f^for males

^g^study found partly beneficial and partly detrimental results

^h^for leisure, but not overall life

#### General health

Three longitudinal studies reported on the relationship between TWFH and the general health of employees. A study by Henke et al. [36], where employee health was based on several underlying health related risk factors (Edington score), showed that those TWFH had overall less risk of developing bad health compared to those who worked from the office. Reusche [28] and Kröll & Nuesch [27] did not find any significant relationship between partly or fully TWFH and self-reported general health. NOS evaluation rated all three studies as good. The collected body of evidence (GRADE) was considered low.

#### Pain

Two cross-sectional studies reported on the relationship between TWFH and general pain. A study by Song & Gao [29] found no association between TWFH (normal hours on weekdays) and pain. Gimenez-Nadal and colleagues [30] found that males TWFH reported significantly lower levels of pain. This association was not found for females. NOS evaluation rated one study as fair [30] and one study as good [29]. The collected body of evidence (GRADE) was considered very low.

#### Well-being

Three cross-sectional [29, 30, 32] and one longitudinal study [39] reported on how TWFH was related to well-being or factors closely linked to well-being. Anderson et al. found a higher degree of positive affective well-being and a lower level of negative affective well-being [32] on days where workers engaged in TWFH, compared to days working at the office. Similarly, Shepherd-Banigan et al. [39] found that TWFH was related to less symptoms of depression in women with young children (≤24 months) who returned to work within 6 months after childbirth. Song & Gao [29] found few associations between TWFH and feeling happiness, sadness, and meaningfulness, but showed a small reduction in happiness for mothers. Another study investigating happiness and sadness, did not find a relationship between TWFH and these feelings [30]. NOS evaluation rated two studies as good [29, 39], one as fair [30], and one as poor [32]. The collected body of evidence (GRADE) was considered very low.

#### Stress

Five cross-sectional [29–31, 33, 35] and one longitudinal quasi-experimental field study [34] investigated the relationship between TWFH and stress. Fonner & Roloff [35] reported that TWFH reduced stress levels. Similarly, Delanoije & Verbruggen [34] showed that workers allowed to telework partly from home reported lower stress on home days, but they found no difference in stress levels between those teleworking partly at home and those working only at the office. Reduced stress when TWFH was also shown by Gimenez-Nadal et al. [30], but only for male workers, and by Baard & Thomas [33], where this relationship was influenced by the number of dependents at home. On the contrary, one study found that TWFH was associated with higher stress levels [29], and another found no significant relationships between TWFH and stress [31]. NOS evaluation rated three studies as good [29, 31, 34], one as fair [30], and two studies as poor [33, 35]. The collected body of evidence (GRADE) was very low.

#### Exhaustion and burnout

Five cross-sectional [29–31, 37, 38] and one longitudinal study [40] investigated the relationship between TWFH and exhaustion (three studies), tiredness (two studies), or burnout (one study). Sardeshmukh et al. found that TWFH generally led to lower degree of exhaustion and indicated the relationship could be mediated by role conflict, role ambiguity, time pressure, support, feedback, and autonomy [38]. Reduced tiredness for home workers was also found by Gimenez-Nadal et al., but only for males [30]. The study by Windeler et al. [40], indicated that TWFH part-time attenuated the detrimental effect increased interpersonal interaction had on exhaustion, but at the same time increased the level of exhaustion seen associated to external interaction. Hoffmann et al. [37] did report that having negative experiences with TWFH, was associated with burnout. The study by Song & Gao [29] found no association between TWFH and tiredness. Similarly, Vander Elst and colleagues [31] found no direct association between TWFH and emotional exhaustion; however, results indicated an indirect relationship via level of felt social support. NOS evaluation rated three studies as good [29, 31, 40], one as fair [30], and two studies as poor [37, 38]. The collected body of evidence (GRADE) for an association between TWFH and exhaustion and burnout was very low.

#### Satisfaction with overall life and leisure

One longitudinal study found no evidence for a relationship between TWFH and leisure satisfaction [27]. Another longitudinal study by Reuschke [28] did find a relationship between TWFH and leisure satisfaction, but not with overall life satisfaction. NOS evaluation rated the two studies as good, and the collected body of evidence (GRADE) for an association between TWFH and satisfaction with overall life and leisure was considered low.

#### Sensitivity analysis

Focusing on results from longitudinal studies rated as “good”, one outcome category has no such studies (pain), three of the outcome categories has one such study each (well-being, stress, exhaustion & burnout), and the two categories with more than one study (general health, satisfaction with life and leisure) show contradicting results. For cross-sectional studies rated as “good”, two categories have no such studies (general health, satisfaction life & leisure) and two outcome categories have only one study each (pain, well-being). Moreover, the two studies for stress show contradicting results and the two studies on exhaustion & burnout claims no association. Combining results from all good studies, irrespective of design, do not provide clear evidence for any associations. Overall, studies rated as poor or fair more often report associations. See Table 3.

## Discussion

In this review, we aimed to systematically select and summarize the up-to-date, available evidence on the potential relationship between TWFH and employee health. Overall, we included 14 studies from five different countries, where nine studies originated from USA. Studies were published from 2010 to 2020 and investigated six different categories of health outcomes. There was considerable heterogeneity in measurement methods of exposure and outcome between studies, and a predominance of cross-sectional studies and/or additional methodological issues. As a result, the body of evidence for the detected outcome categories received a GRADE-score of low or very low, with the conclusion being the discovery of a significant knowledge gap.

To our knowledge there are no other systematic reviews recently published with up-to-date evidence on the relationship between TWFH and employee health. Two reviews by de Macêdo et al. [41], and Charalampous et al. [42], reported on well-being as outcome, but included all types of teleworkers, not differentiating between TWFH and other remote locations (another company site, hotel, airport etc.). Both reviews found positive and negative aspects with telework but indicated an overall beneficial impact on well-being. Buomprisco et al. [43] recently presented some possible health implications with non-specified telework in their review, but did not provide summary tables, nor did they report on important issues such as a detailed search strategy, selection criteria and selection process, data extraction, or quality assessment. A newly published rapid review by Oakman and colleagues [44] investigated the mental and physical health effects of TWFH and concluded there was a limited number of studies available, often with conflicting results. Thus, there is agreement between their rapid review and the present systematic review, despite some differences in methodology (e.g. search years for publications, included study types, restrictions towards TWFH exposure, and restrictions towards impact of COVID-19).

### Main limitations of the available research

Eight out of the 14 included studies had cross-sectional design, making it impossible to conclude on causality (e.g. one cannot distinguish between the occurrence of an employee health issue that leads to an TWFH arrangement and a health issue caused by TWFH arrangements). Randomized-controlled studies or other types of intervention studies with reasonable follow-up would be helpful and feasible to increase knowledge on the relationship between TWFH and employee health.

While acknowledging that some important health issues are subjective, it is a limitation that all exposures and reported outcomes in the included studies were based on self-reports, and in many cases not standardized or validated. Further, even though all are using self-reports, some studies use single item measures reflecting global evaluations of a health outcome (e.g. overall perceived health [27]), whereas others include several more specific aspects of health (e.g. happiness, sadness, tiredness) that are assumed to contribute to a health outcome of interest (e.g. well-being) [29–31]. Again, others provide several specific aspects of health with an additional analysis calculating the combined estimate for this set of factors, which then is represented as the health outcome of interest [32, 36]. In this systematic review we consequently presented the combined estimate when it was given, and otherwise split results for specific factors into fitting outcomes categories. Additionally, different studies often conceptualized and defined both TWFH and health outcomes in different ways.

Nine out of 14 included studies originated from the USA, some with partly overlapping samples. Over half of the investigated participants were working in the American labor market. Due to major dissimilarities between e.g. labor markets, environment, and socioeconomics in different countries, this severely limit the potential for generalization of results.

Our search strategy opened for any type of health-related outcome. Considering this, the most important limitation of the available research is that studies carried out on a wide range of relevant, important, and plausible health outcomes are lacking. Despite the wide search strategy, we identified no studies pertaining to important, plausible health challenges such as: mood and anxiety disorders defined by clinical criteria; hypertension (cardiovascular disease); sleep disturbance; workability (risk of sick leave & disability pension).

### Implications of TWFH and health

Two out of the four included studies carried out on well-being included in the present review indicated that TWFH may have an advantageous effect on this outcome category. Still, only one of these two studies was of good quality and with a longitudinal design, with its beneficial result only fund for working women with children aged ≥2 years. For the outcome categories stress, and exhaustion and burnout the studies rated with poor and fair quality, suggested a beneficial relationship, while the studies rated good quality indicated no association or detrimental association. Further, we found limited and divergent results for the relationships between TWFH and the employee’s life and leisure satisfaction and general health.

In all, we need to be very precautious when discussing the implication of these results, considering the overall quality and the limited number of studies forming the body of evidence. However, if TWFH could reduce stress and exhaustion and increase the feeling of well-being in employees, employers may utilize this work arrangement to affect these aspects, either in specific situations or permanently. With that said, it is also possible that employees’ individual characteristics may affect the degree to which TWFH is advantageous. For example, it is suggested that individuals with a greater need of social interaction and stimulus from the surrounding environment or lacks a social network outside of work, will be more negatively affected by TWFH compared to those not fitting these descriptions [32]. Moreover, those with high openness to experience may cope better with the implementation of new ways of working [32].

Findings also suggest that family situation, facilities/housing situation, being a provider/taking care of a child or elderly may be of importance considering the relationship between TWFH and health. Hoffmann and colleagues reported that negative experiences with TWFH most commonly were seen in connection to family and/or children related issues or technological problems [37]. Shepherd-Banigan et al. [39] did find that for working women additionally caring for child, TWFH had an advantageous impact on depression symptoms, and indicated the gain was not in the number of hours TWFH, but the possibility to do so when needed. It is previously suggested that balance between work and family may have impact on employee health [9, 45], and that flexibility in where and when an individual works may contribute to this balance [10, 46]. Telework is commonly associated with increased autonomy and may therefore reduce feeling of overload and work-related stress [47]. On the other hand, TWFH may also blur lines between work and private life, or trigger the feeling of never leaving work, which may undermine the autonomy aspect and have detrimental effects on health [48, 49]. For instance, Song & Gao [29] found increased stress among fathers- and reduced happiness among mothers TWFH, and Windeler et al. [40] found higher levels of exhaustion in connection to TWFH in older workers and males. It is possible that TWFH arrangements could affect also other private life matters, like physical activity (PA). The impact could be both detrimental and beneficial and act out through changes in e.g. active commuting or leisure time available for PA, which is known to have impact on health and sick leave [50]. Further, one should also be aware to which degree TWFH would change the content of a certain job. While some jobs may be very compatible to such work arrangements, others would be less compatible or not compatible at all. Introducing TWFH to jobs not fully compatible could lead to worker frustration and a feeling of insufficiency [51].

Finally, when evaluating TWFHs relationship to employee health there seems also necessary to be aware of both voluntariness, intensity, and the type of TWFH considered. As an example, it is indicated that reported stress may be affected differently when the work is carried out within normal working hours, outside normal working hours (overtime, weekends, holiday), or if work is brought home after a normal workday at the office [29].

Given the plausible influences of a multitude of mediating and moderating factors, the need for more research is evident. Generally, the relations seen with TWFH are likely to be complex, and knowledge concerning job content and each employee and their overall life situation, balanced with the collective bargaining at company level could guide decisions on TWFH arrangements [51].

### Limitations of this systematic review

We chose not to include studies investigating exposure to unspecified remote work, since other types of remote work might have different characteristics than TWFH arrangement. Thus, there is a possibility that some relevant studies were not included. In some studies, it was also difficult to decide if the exposure was TWFH or involved also other types of remote work. We further chose to focus on normal working hours, despite some publications investigated TWFH in relation to overtime, weekends, bringing work home etc.

We restricted our search to studies published in 2010 or more recent, which means that studies published prior to this were not included. This was a reasonable, but arbitrary set date, with the purpose of selecting up-to-date studies, due to the rapid change in technology also changing the format of TWFH. To ensure the evidence from this review reflected a normal TWFH situation, we further chose not to include studies carried out under the COVID-19 pandemic where we additionally believed specific restrictions in relation to the pandemic were likely to affect the results more than the exposure to TWFH. In making these decisions we needed to exercise some level of subjective judgement, which implies the possibility that different decisions might have been made by other research groups.

To evaluate the body of evidence for outcomes, we constructed outcome categories. Some of these outcome categories included outcomes that were somewhat different, which may have contributed to heterogeneity in the outcome categories. Still, it made sense to group outcomes of similar constructs with a purpose of evaluating the body of evidence in a domain, instead of merely reporting individual outcomes separately. The number of studies in each outcome category was too small to make a sensible establishment or refusion of publication bias. However, unpublished studies would be less likely to have statistically significant results and be more likely to have smaller effect estimates than published studies [52]. Since our review generally found very limited evidence for associations between TWFH and health, these results are less likely to be altered by plausible unpublished studies.

### Strengths of this systematic review

This is the only systematic review of recent date assessing the evidence on the relationship between TWFH and employee health, provided in a time where important decisions are necessary to be made regarding the role of TWFH in future work arrangements. We welcomed all health-related outcomes and did not exclude based on specific preset outcomes. Thus, we provide a broad overview of relevant, available studies on how TWFH may affect any aspect of employee health. The systematic review followed recommended guidelines for planning, execution, and reporting of a systematic review.

### Further research

This review reveals a paucity of knowledge on the relationship between TWFH and a vast variety of health outcomes, which is of critical importance to elucidate when facing a plausible post-pandemic up-scaled implementation of TWFH arrangements. Researchers should be proactive towards enterprises interested in testing TWFH and increasing knowledge on TWFH arrangements in their businesses. When possible, researchers should aim to carry out interventions with randomized allocation. In this setting one should also consider the impact of characteristics like e.g. gender, family situation, and type of job in order to do solid between-groups comparisons. Clear definitions of the type of telework that is being investigated is crucial, with accompanying precise and valid measures for quantification.

## Conclusion

This systematic review systematically investigated the available research recently published on the association between TWFH and health. Overall, there were few studies investigating a limited number of outcomes, especially when considering the huge number of potential outcomes of importance in this setting. Additionally, many studies had suboptimal designs and/or additional methodological issues, resulting in the body of evidence for the discovered outcomes receiving either low or very low GRADE scores. Thus, there is a paucity of high-quality and up-to-date research on how TWFH affects employee health.

## Supplementary Information

**Additional file 1: Supplementary S1.** Search strategy

**Additional file 2: Supplementary S2.** Thresholds for converting the NOS scales to AHRQ standards (good, fair, and poor).

**Additional file 3: Supplementary S3.** Outcomes reported by studies and the respective outcome category they were placed.

## Data Availability

All relevant data is included in the manuscript or supplementary.
