# Correction: The relationship between telework from home and employee health: a systematic review

**DOI:** 10.1186/s12889-022-13334-2

**Published:** 2022-05-10

**Authors:** Lars-Kristian Lunde, Lise Fløvik, Jan Olav Christensen, Håkon A. Johannessen, Live Bakke Finne, Ingrid Løken Jørgensen, Benedicte Mohr, Jolien Vleeshouwers

**Affiliations:** 1 Department of Occupational Medicine and Epidemiology, National Institute of Occupational Health, Oslo, Norway; 2 Department of Work Psychology, National Institute of Occupational Health, Oslo, Norway

**Correction to: BMC Public Health 22, 47 (2022)**

**https://doi.org/10.1186/s12889-021-12481-2**

The original publication of this article [1] contained 2 errors:

1. The PRISMA Flow diagram (Fig. 1) contained incorrect values
2. The following record-values were incorrect:
  - 298 (Full-text articles assessed for eligibility) should be 289
  - 53 (Studies included in qualitative synthesis) should be 50
  - 39 (Studies reflecting only work environment outcomes) should be 36

The original article has been updated to correct these errors. This does not impact the conclusions of the article.
