# Supplementary material for: The relationship between telework from home and employee health: a systematic review
Source: BMC Public Health. 2022 Jan 7;22:47. doi: 10.1186/s12889-021-12481-2 (PMC8741267; doi:10.1186/s12889-021-12481-2)
Supplement: Supplementary file 1 — Additional file 1: Supplementary S1. Search strategy [file 12889_2021_12481_MOESM1_ESM.pdf]

## Search strategy – Working from home:

**Scopus:**

TITLE-ABS ( "working from home" ) OR TITLE-ABS ( "working at home" ) OR TITLE-ABS ( "remote work*" ) OR TITLE-ABS ( "Home-Based Work*" ) OR TITLE-ABS ( "virtual office" ) OR TITLE-ABS ( "virtual work" ) OR TITLE-ABS ( telecommuting ) OR TITLE-ABS ( telework* ) OR TITLE-ABS ( telehomework* ) OR TITLE-ABS ( "mandatory work" ) OR TITLE-ABS ( "home-working" ) OR TITLE-ABS ( homeworking ) OR TITLE-ABS ( "home working" ) AND ( LIMIT-TO ( PUBSTAGE , "final" ) ) AND ( LIMIT-TO ( DOCTYPE , "ar" ) OR LIMIT-TO ( DOCTYPE , "re" ) ) AND ( EXCLUDE ( SUBJAREA , "ENGI" ) OR EXCLUDE ( SUBJAREA , "COMP" ) OR EXCLUDE ( SUBJAREA , "MATH" ) OR EXCLUDE ( SUBJAREA , "MATE" ) OR EXCLUDE ( SUBJAREA , "PHYS" ) OR EXCLUDE ( SUBJAREA , "ENER" ) OR EXCLUDE ( SUBJAREA , "EART" ) OR EXCLUDE ( SUBJAREA , "CENG" ) OR EXCLUDE ( SUBJAREA , "AGRI" ) OR EXCLUDE ( SUBJAREA , "BIOC" ) OR EXCLUDE ( SUBJAREA , "CHEM" ) OR EXCLUDE ( SUBJAREA , "NEUR" ) OR EXCLUDE ( SUBJAREA , "PHAR" ) OR EXCLUDE ( SUBJAREA , "IMMU" ) OR EXCLUDE ( SUBJAREA , "DENT" ) OR EXCLUDE ( SUBJAREA , "VETE" ) ) AND ( LIMIT-TO ( PUBYEAR , 2021 ) OR LIMIT-TO ( PUBYEAR , 2020 ) OR LIMIT-TO ( PUBYEAR , 2019 ) OR LIMIT-TO ( PUBYEAR , 2018 ) OR LIMIT-TO ( PUBYEAR , 2017 ) OR LIMIT-TO ( PUBYEAR , 2016 ) OR LIMIT-TO ( PUBYEAR , 2015 ) OR LIMIT-TO ( PUBYEAR , 2014 ) OR LIMIT-TO ( PUBYEAR , 2013 ) OR LIMIT-TO ( PUBYEAR , 2012 ) OR LIMIT-TO ( PUBYEAR , 2011 ) OR LIMIT-TO ( PUBYEAR , 2010 ) ) AND ( LIMIT-TO ( LANGUAGE , "English" ) OR LIMIT-TO ( LANGUAGE , "German" ) )

**PubMed:**

((((((((((("working from home"[Text Word]) OR ("working at home"[Text Word])) OR ("remote work*"[Text Word])) OR ("Home-Based Work*"[Text Word])) OR ("virtual office"[Text Word])) OR ("virtual work"[Text Word])) OR (telecommuting[Text Word])) OR (telework*[Text Word])) OR (telehomework*[Text Word])) OR ("mandatory work"[Text Word])) OR ("home-working"[Text Word])) OR (homeworking[Text Word])) OR ("home working"[Text Word])

**Ovid-basene: (Medline, Embase, Amed and PsycINFO):**

Database: AMED (Allied and Complementary Medicine) <1985 to September 2020>, Embase <1974 to 2020 September 22> , Ovid MEDLINE(R) and Epub Ahead of Print, In-Process & Other Non-

Indexed Citations, Daily and Versions(R) <1946 to September 21, 2020>, APA PsycInfo <1806 to September Week 2 2020>

Search Strategy:

--------------------------------------------------------------------------------

1 work* from home.tw. (5914)

2 work* at home.tw. (1012)

3 (remote* adj2 work*).tw. (1238)

4 Home-Based Work.tw. (78)

5 (home adj2 workspace*).tw. (4)

6 virtual office.tw. (90)

7 virtual work.tw. (328)

8 telework*.tw. (553)

9 telecommuting.tw. (391)

10 telehomework*.tw. (5)

11 mandatory work.tw. (65)

12 homeworking.tw. (19)

13 home working.tw. (123)

14 1 or 2 or 3 or 4 or 5 or 6 or 7 or 8 or 9 or 10 or 11 or 12 or 13 (8500)

15 14 and 2010:2021.(sa_year). (4866)

16 limit 15 to (danish or english or norwegian or swedish) (4657)

17 limit 16 to human [Limit not valid in AMED; records were retained] (4026)

18 limit 17 to (journal article or "review" or thesis or article or article in press or adaptive clinical trial or case reports or clinical study or clinical trial, all or clinical trial or comparative study or controlled clinical trial or "corrected and republished article" or introductory journal article or meta analysis or observational study or pragmatic clinical trial or randomized controlled trial or "systematic review" or systematic reviews as topic or validation study) [Limit not valid in AMED,Embase,Ovid MEDLINE(R),Ovid MEDLINE(R) Daily Update,Ovid MEDLINE(R) In-Process,Ovid MEDLINE(R) Publisher,APA PsycInfo; records were retained] (3017)

19 limit 18 to journal [Limit not valid in AMED,Ovid MEDLINE(R),Ovid MEDLINE(R) Daily Update,Ovid MEDLINE(R) In-Process,Ovid MEDLINE(R) Publisher; records were retained] (3009)

20 (Virtual Reality or Dermatitis or arthritis or military or veteran or veterinarian).tw. (753535)

21 19 not 20 (2937)

22 remove duplicates from 21
