# Supplementary material for: The relationship between telework from home and employee health: a systematic review
Source: BMC Public Health. 2022 Jan 7;22:47. doi: 10.1186/s12889-021-12481-2 (PMC8741267; doi:10.1186/s12889-021-12481-2)
Supplement: Supplementary file 2 — Additional file 2: Supplementary S2. Thresholds for converting the NOS scales to AHRQ standards (good, fair, and poor). [file 12889_2021_12481_MOESM2_ESM.docx]

**Supplementary S2 Thresholds for converting the NOS scales to AHRQ standards (good, fair, and poor)**

**Good quality:** 3 or 4 stars in the selection domain AND 1 or 2 stars in comparability domain AND 2 or 3 stars in the outcome/exposure domain

**Fair quality:** 2 stars in the selection domain AND 1 or 2 stars in comparability domain AND 2 or 3 stars in the outcome/exposure domain

**Poor quality:** 0 or 1 star in the selection domain OR 0 stars in comparability domain OR 0 or 1 star in the outcome/exposure domain
