# Supplementary material for: The relationship between telework from home and employee health: a systematic review
Source: BMC Public Health. 2022 Jan 7;22:47. doi: 10.1186/s12889-021-12481-2 (PMC8741267; doi:10.1186/s12889-021-12481-2)
Supplement: Supplementary file 3 — Additional file 3: Supplementary S3. Outcomes reported by studies and the respective outcome category they were placed. [file 12889_2021_12481_MOESM3_ESM.docx]

| **Study** | **General health** | **Pain** | **Well-being** | **Stress** | **Exhaustion & Burnout** | **Satisfaction life & leisure** |
| --- | --- | --- | --- | --- | --- | --- |
| Anderson et al. 2015 |  |  | Negative affective WB  Positive affective WB |  |  |  |
| Baard & Thomas 2010 |  |  |  | Stress |  |  |
| Delanoeije & Verbruggen 2020 |  |  |  | Stress |  |  |
| Fonner & Roloff 2012 |  |  |  | Stress from interuptions |  |  |
| Giménez-Nadal et al. 2020 |  | Pain | Happiness  Sadness | Stress | Tiredness |  |
| Henke et al. 2016 | Edington risk score |  |  |  |  |  |
| Hoffman et al. 2020 |  |  |  |  | Burnout |  |
| Kroll & Nüesch 2019 | Perceived health |  |  |  |  | Leisure satisfaction |
| Reusche D. 2019 | Satisfaction with health |  |  |  |  | Satisfaction with life overall  Satisfaction with leisure time |
| Sardeshmukh et al. 2012 |  |  |  |  | Exhaustion |  |
| Shepherd- Banigan et al. 2016 |  |  | Symptoms of depression |  |  |  |
| Song & Gao 2020 |  | Pain | Happiness  Sadness  Meaningfulness | Stress | Tiredness |  |
| Vander Elst et al. 2017 |  |  |  | Cognitive stress | Emotional exhaustion |  |
| Windeler et al. 2017 |  |  |  |  | Work exhaustion |  |
| **Total studies/total outcomes** | 3/3 | 2/2 | 4/8 | 6/6 | 6/6 | 2/3 |

**Supplementary S3. Outcomes reported by studies and the respective outcome category they were placed.**
